# Poisoning Attacks against Communication and Computing Task Classification and Detection Techniques

**DOI:** 10.3390/s24020338

**Published:** 2024-01-05

**Authors:** Younes Salmi, Hanna Bogucka

**Affiliations:** Institute of Radiocommunications, Poznan University of Technology, 61-131 Poznan, Poland; hanna.bogucka@put.poznan.pl

**Keywords:** data poisoning, k-means algorithm, k-nearest neighbors algorithm, clustering, edge computing

## Abstract

Machine learning-based classification algorithms allow communication and computing (2C) task offloading from the end devices to the edge computing network servers. In this paper, we consider task classification based on the hybrid k-means and k′-nearest neighbors algorithms. Moreover, we examine the poisoning attacks on such ML algorithms, namely noise-like jamming and targeted data feature falsification, and their impact on the effectiveness of 2C task allocation. Then, we also present two anomaly detection methods using noise training and the silhouette score test to detect the poisoned samples and mitigate their impact. Our simulation results show that these attacks have a fatal effect on classification in feature areas where the decision boundary is unclear. They also demonstrate the effectiveness of our countermeasures against the considered attacks.

## 1. Introduction

Radio communication technology has continuously been advancing for the past few decades. Recent developments such as fifth-generation (5G) mobile radio communication promise to take connectivity to new heights. In comparison with their predecessors, 5G and 6G wireless communications deliver faster speeds, lower latency, higher reliability, and increased capacity for anticipated services. The increased bandwidth and reduced latency of 5G enable a wide range of applications, including autonomous cars, robotics, virtual reality, and the Internet of Things (IoT). It promotes the establishment of these new values by enabling the development of new services in three major use case domains: enhanced mobile broadband (eMBB), ultra-reliable low-latency communication (URLLC), and massive machine-type communications (mMTC).

While 5G is already pushing the boundaries of wireless communication, researchers, engineers, and industry practitioners have begun to look beyond it. Beyond 5G, wireless network development, often called sixth generation (6G) or beyond 5G, aims to break down barriers and explore new frontiers. One of the primary goals of wireless communication beyond 5G is to push the boundaries of latency, dependability, connection, and coverage. Although the specifics of the technology are now speculative, numerous critical areas have emerged as possible priority areas for future wireless communication system development.

According to the recent Ericsson Mobility Report [1], 5 billion 5G mobile subscribers will be operating by 2028. Furthermore, wireless communication of 34.7 billion machines and devices is predicted to compose the IoT by that year. This expected massive human-machine communication means tremendous data flow across communication channels. Moreover, emerging applications such as intelligent healthcare, intelligent transportation, and interactive gaming impose more demanding quality of service (QoS) requirements. Edge computing is emerging as a unique computing paradigm that leverages cloud computing and pushes it closer to the edge to deal with these challenges.

The development of edge-computing networks may lead to the reduction of communication latency since the networking distance between the end devices and processing (computing) nodes will be shortened. It will also enable local data processing, which is critical for applications requiring real-time decision making and instant reaction. Furthermore, rather than burdening the main network or cloud data centers with all computing requirements, the edge nodes can execute localized jobs and alleviate the central infrastructure, a practice known as task offloading.

To fully benefit from edge computing, edge intelligence using artificial intelligence (AI) and machine learning (ML) is an essential paradigm. For example, optimal communication and computing (2C) task offloading to the appropriate servers is a challenging problem, and its optimization is not always possible, given the limited knowledge of the network components at the edge. Therefore, ML can be used to classify and predict the generated 2C requests. This classification is intended to support the near-optimal delegation of a request to an appropriate server at the edge of a network.

To enable the execution of edge intelligence apps and services, the mobile edge host in the future 5G or 6G architecture operates a mobile edge platform. The 5G or 6G network design will be almost totally virtualized and dependent on the software features. As a result, it is open to being utilized, attacked, and interrupted by hackers. In the case of sensitive applications, such as mission-critical ones that need URLLC service, attacks against ML algorithms used for traffic steering may result in disastrous failures. In the edge-computing network under consideration, 2C tasks should be classified based on service performance requirements (for example, end-to-end latency, packet error rate (PER), or the computational complexity of a delegated task). This in turn would enable 2C task delegation to the preferred edge-computing machine or server characterized by the delay associated with the transmission to this server, queuing and computing clock frequency, packet error probability associated mostly with transmission and networking, and offered computational power.

The classification criteria may alter, but in the case of multi-class classification with set sensitivity levels (from noncritical to highly critical actions), any misclassification might result in a fatal classification error. Adversarial machine learning (AML) [2] is a term describing the study of the attacks on ML algorithms and the defenses against such attacks. Attackers can use a variety of tactics to target a system (see Table 1).

### 1.1. Scope

This study explores the impact of artificial intelligence security threats on 2C task classification for edge computing. The research will involve a scientific analysis of the effects of poisoning attacks on intelligent 2C task classification. As a result, wireless distortions (e.g., fading) are not considered. This study will also assess the countermeasure solutions to such threats.

### 1.2. Originality

This work is original since it addresses intelligent 2C task classification and the security threats against it. First, to the best of our knowledge, this is the first work that addresses AI assistance to task classification for both communication and computing parts. Second, the applications and realization of poisoning attacks and AML threats in general are poorly covered for wireless communications. Finally, the study of a hybrid k-means and k-NN ML application technique, poisoning threats, and anomaly detection countermeasures using statistical tools makes this initial study unique.

### 1.3. Methodology

Conducting a scientific investigation on poisoning attacks requires a simulation or implementation of the targeted application. The simulation should follow a specific protocol. The methodology that was followed in this paper incorporates six steps: first, define the problem intended for simulation; second, formulate the model; third, test the model, and compare its behavior with the behavior of the actual problem; fourth, identify and collect the data needed to test the model; fifth, run the simulation; and sixth, analyze the results of the simulation.

### 1.4. Contributions

Throughout this article, we propose a machine learning technique based on a hybrid of k-means clustering and k-nearest neighbors to assist with near-optimal 2C task classification. In addition, two poisoning attacks will be discussed. These attacks are designed to fool the ML classifier. The effects of these attacks on the ML classification system will be briefly shown. Furthermore, two mitigation techniques based on anomaly detection will be assessed.

## 2. Literature Review and Paper Contributions

### 2.1. Classification

Several approaches are designed to address task allocation in edge computing. Some use a centralized controller that controls the entire network. Others proposed decentralized techniques that allow each node in the network to allocate independently. Hybrid techniques use the advantages of both of the previous approaches [3,4,5,6]. However, these techniques can be energy-exhaustive and suboptimal due to the lack of global information about the network. The idea behind addressing the communication part comes from the analysis of the energy consumption of such systems. As a result, researchers started to focus on the communication and computing parts jointly [7,8]. However, given the limited knowledge of the network components at the edge, optimal communication and computing (2C) task allocation to the appropriate servers is a challenging problem.

Clustering as unsupervised learning is utilized for discovering the patterns of the data [9,10,11]. This can be used to assist near-optimal 2C task classification. Clustering algorithms such as density-based spatial clustering of applications with noise (DBSCAN) were extensively used for general data clustering [12,13,14,15,16,17,18,19,20,21]. However, these techniques are computationally expensive for large datasets. They may thus fail to satisfy the ultra-low-latency requirement from the communication perspective and the computational efficiency requirement from the computing perspective. A quick and efficient technique for huge datasets that addresses this issue is *k-means* [22]. This technique was used for task classification in edge computing [23]. But, implementing *k-means* on 2C classification data with non-spherical clusters is impracticable. Furthermore, the technique requires an initial cluster number (k), and the choice can be far from the optimal k value. As a result, extra tools to define the optimal k value must be considered. In addition, the initial centroids are chosen randomly from the dataset, and with different combinations, the clustering results can be different. Moreover, because the clustering scheme will not change after a few new examples in the dataset, it is impractical to classify each new request using the same process.

As a result, these new requests will be predicted using a less complex approach. Supervised learning techniques, as they are less complex techniques, are a good choice [24,25]. However, the chosen approach must be adaptable to *k-means* modifications after each individual epoch, and the lack of a training dataset restricts the considerations to non-training phase techniques. The choice in this case is k′-nearest neighbors (*k′-NN*) [26], where new requests are assigned to the nearest cluster based on a metric.

The method allocates each example based on the neighbors’ majority voting, although the technique is sensitive to the number of k′ neighbors. If k′ is reduced, then *k′-NN* may overfit the *k-means* data. Higher k′ values, on the other hand, lead to greater computing complexity.

### 2.2. Poisoning Attacks

In recent years, the data poisoning literature has addressed attacks in a range of scenarios as well as diverse applications, with threat models ranging from attackers having only data access to attackers controlling the entire training process. Poisoning attacks were extensively discussed for other scenarios concerning computer vision (CP) [27,28,29] and natural language processing (NLP) [30,31,32]. In general, AML in wireless communications is still poorly explored, although adverse wireless channels and introduced distortions are inherent in mobile radio communication.

Some works discussed the topic in a general way that can be applied to several classification problems. For example, in [33], the authors discussed their novel poison threat algorithm to generate adversarial examples that deceive ML classifiers such as k-means and spatial clustering. Another proposed work [34] dealt with fair ML classification under these attacks and the high poisoning vulnerability of this classifier.

To launch the attacks against 2C task classification, a variety of techniques can be borrowed from wireless research works. Some of them are conventional wireless attacks. Jamming, for example, might prevent an ML model from being trained correctly on data provided by a given source. This strategy is used to change some training examples, resulting in data poisoning [35]. Another example would be wireless medium eavesdropping to obtain model features, resulting in stealing the trained model or jeopardizing data privacy. In [36], the authors considered class sniffing and quantity inference attacks. These attacks find the class (the label) of a certain example and determine the composition proportion of the training labels owned by selected clients, respectively.

It is worth mentioning that a large group of works consider attacks on ML algorithms using other ML algorithms or hybrid attacks (conventional or ML attacks). One recent work [37] discussed generative adversarial networks (GANs) in 5G communication systems. AML attacks on wireless communication are categorized and summarized in [38].

### 2.3. Paper Contribution

To the best of our knowledge, this paper is the first work that addresses poisoning attacks against 2C task classification. Our paper is different because, first of all, it sets the whole problem in the edge-computing scenario and shows the implications of these attacks and methods in wireless communication and edge computing. Second, in contrast to the other works that use optimization methods for 2C task offloading, we use more practical, ML-based tools. Moreover, the existing mitigation techniques such as anomaly detection [39] and adversarial training [40] abstract from the specifics of wireless communication and 2C task classification problems. In this paper, we investigate how data poisoning attacks impact 2C task classification and offloading and how they can be mitigated.

The work starts with an introduction of our considered system model. Then, a hybrid ML technique is proposed to assist the near-optimal 2C classification. Next, two radio communication-specific poisoning attacks are considered and described. The effects of these attacks on 2C task classification are analyzed. In the last step, poisoning mitigation techniques are considered that can detect these attacks. Their effects on the classification system are shown.

## 3. System Model

### 3.1. System Architecture

**Figure 1:**
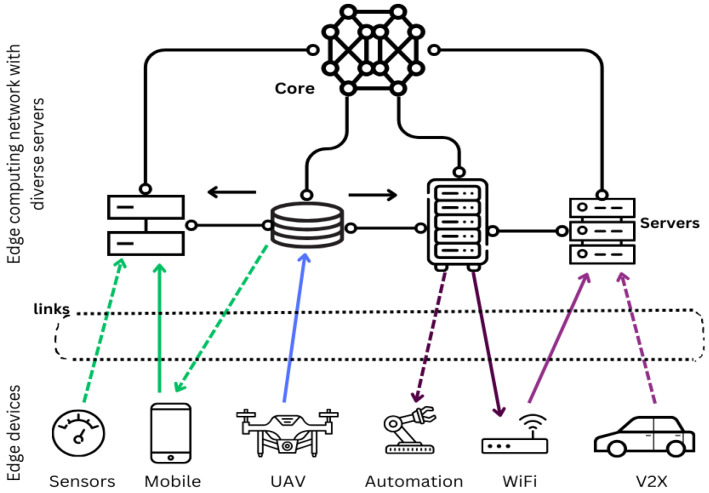
System architecture.

The considered edge-computing network is an edge-computing network that consists of diverse servers (with diverse computing and storage capabilities) and diverse IoT devices (generating diverse communication and computing requests). The network architecture is presented in Figure 1.

In this work, the 2C tasks (requests) are represented by three features: (1) the required end-to-end latency in milliseconds, (2) the required reliability (in terms of the packet error rate (PER)), and (3) the computational complexity in floating-point operations $\times 10^{9}$ (GFLOPs). In general, other features might be considered for 2C task classification (although some may translate to the ones given above, such as the task complexion speed). Here, we selected the ones that were most relevant for 2C tasks in 5G and 6G services.

The idea was to classify the tasks to delegate each task to the appropriate server or virtual machine (VM). The allocation might follow several scenarios, but here, for simplicity, every available server or VM and the associated communication link was considered to serve a given requirement (latency, reliability, or task complexity) either with a high or low attribute. Hence, eight servers with associated communication links (called 2C links for simplicity) were possible to consider, which represented eight 2C links, as presented in Table 2. The links’ features are represented by a single binary digit, with zero being for low-quality service and one being for high-quality service. The decision about the state of each 2C link’s feature $x_{i}^{n}$ , n∈{1,2,3} was obtained as follows:

(1) $${x_{i}}^{n}=\left\{\begin{array}{cc} 1(\mathrm{high}) & {x_{i}}^{n}>\alpha_{i}^{n}\\ 0(\mathrm{low}) & \mathrm{otherwise} \end{array}\right.$$

where $\alpha_{i}^{n}$ is the middle point of the *n*th feature’s interval and *i* is the 2C link ID ( i∈1,2,…8 ).

### 3.2. Dataset

The dataset representing the 2C tasks (requests) with the three mentioned features consisted of l=1000 examples. For these 2C requests, some representative cases were chosen.

The communication- and computing-related features are described below with labels as tuples, with latency in terms of [milliseconds, PER]. The communication labels [ $\mu_{j}^{1}$ , $\mu_{j}^{2}$ ] for each use case were obtained according to the QoS of 5G defined by ETSI in “(2022-05)168 3GPP TS 23.501 version 17.4.0” release 17:

- Process automation [50, $10^{-3}$ ].
- V2X collision avoidance [5, $10^{-4}$ ];
- Mission-critical data [200, $10^{-6}$ ];
- Intelligent transportation system [30, $10^{-5}$ ];
- Low latency eMBB [10, $10^{-6}$ ];
- Live streaming [300, $10^{-8}$ ];
- Conversational video [100, $10^{-2}$ ];
- Interactive gaming [100, $10^{-3}$ ].

For the computational complexity feature, random values following a uniform distribution were selected in the range of [200, 5000] GFLOPs. The choice here can be supported by the diversity of requests generated. Video processing or ML model training tasks may have higher complexity than signaling tasks. In addition, the same category of tasks such as video processing, compression, and size modification may have different complexities. Moreover, same-category tasks with the same process, such as compression, may differ in their workloads. All these points make the choice highly random.

The dataset *D* combining all features consisted of 1000 examples with 3 features each. The representative cases were modeled with 2D Gaussian distributions for the first two axes (end-to-end latency and reliability), with the means $\mu^{n}$ and the variances $\sigma^{n}=\sqrt{0.1\times \mu^{n}}$ . For the last axis (computational capability), the dataset was modeled with a single uniform random distribution.

## 4. The Proposed 2C Task Classification Technique

The stochastic nature of the 5G and 6G usage patterns and the unavailable knowledge about the network nodes make classification optimization of the requests challenging. To address this, the focus was on self-learning networks and systems that could autonomously manage resources and control functions.

The method of *k-means* clustering is a simple, iterative unsupervised technique used to partition the dataset into distinct groups of 2C requests or clusters. Each request belongs to the cluster with the closest mean (cluster centers or cluster centroids). This algorithm aims to minimize the within-cluster variance and maximize the between-cluster variance.

Our *k*-means algorithm starts by randomly initializing *k* cluster centroids within the 3D feature space. Then, it assigns each data point to the nearest centroid based on a distance metric, which is the 3D Euclidean distance here. After that, it recalculates the centroids of each cluster by taking the mean of all data points assigned to that cluster. It repeats the assignment step, assigning each data point to the nearest centroid based on the updated centroids. It continues the update and reassignment steps iteratively until convergence is reached.

Convergence is achieved when the centroids no longer move significantly or when a predefined maximum number of iterations is reached. The algorithm converges to a solution where each data point belongs to the cluster with the closest centroid. The *k-means* algorithm seeks to minimize the within-cluster sum of squared distances (inertia) through iterative optimization of the cluster assignments and updating the cluster centroids. It is important to note that the *k-means* algorithm is sensitive to the initial random initialization, and the algorithm may converge to different solutions depending on the starting points.

However, due to the large number of operations performed by the *k-means* algorithm, it is not convenient to repeat the calculations for the arrival of a limited number of tasks. To handle this, a simple algorithm with lower complexity and a training-free process is used. Here, we suggest the use of the *k′ nearest neighbors* algorithm (*k′-NN*), where k′ is a different value from *k*. It focuses on the similarity between the input data and the labeled examples in the dataset.

The assembled algorithm collects a labeled dataset consisting of the input samples (2C requests clustered by *k-means*) and their corresponding class labels (centroids obtained by *k-means*). Then, it receives an unlabeled data point (new incoming 2C tasks) that needs to be classified or predicted. After that, the similarity (distance) between the new request points and all the labeled requests in the dataset is computed. The used distance metric is the same as the one used in *k-means*) (i.e., the 3D Euclidean distance).

The next step of the algorithm is voting; in other words, *k′-NN* selects the k′ nearest neighbors to the input data point based on the calculated similarity, and then it determines the class label of the input data point by majority voting among the class labels of the k′ nearest neighbors. The algorithm returns the predicted class label or the target value for the new data point.

**Figure 2:**
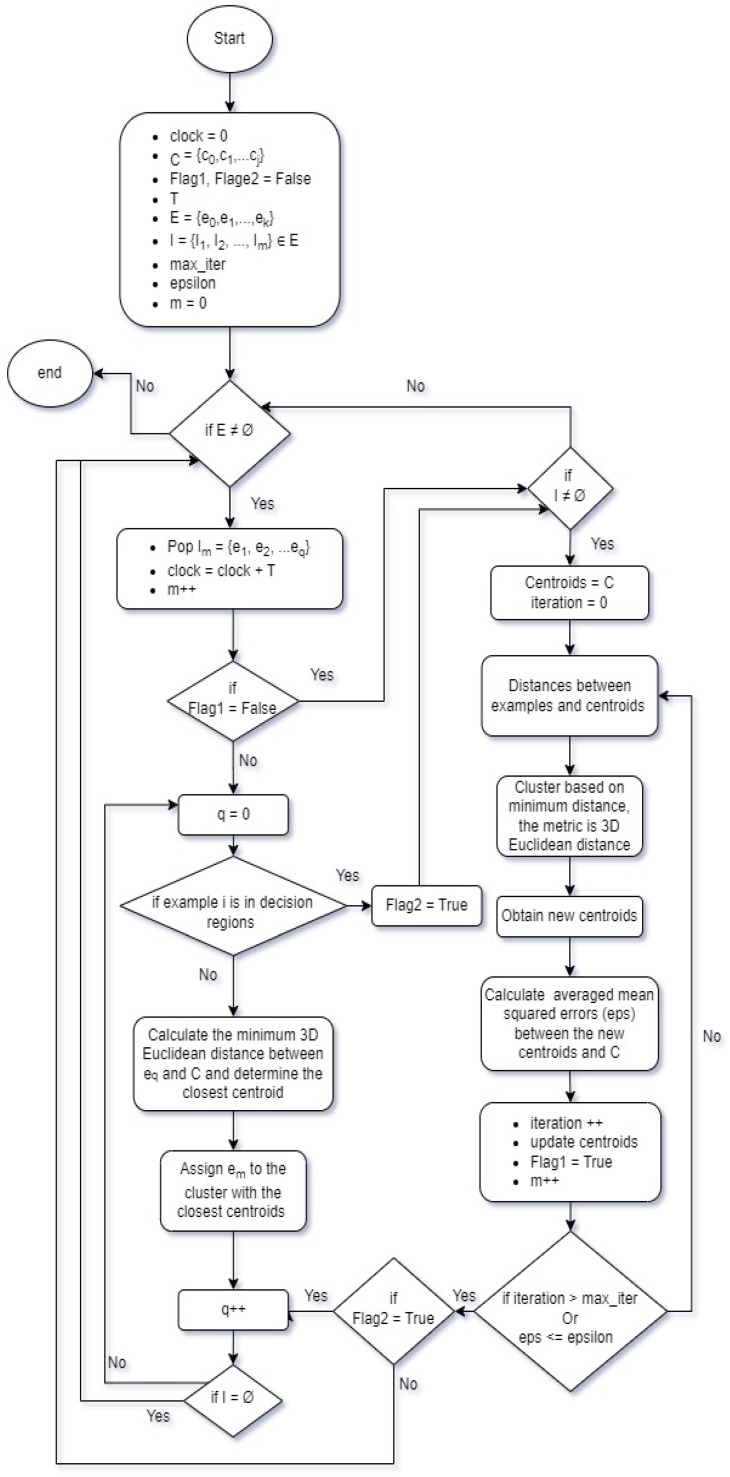
Hybrid *k-means* and *k′-NN* algorithm.

Assembling both techniques leads to a hybrid *k-means/k′-NN* technique described by the flowchart in Figure 2. Where E is the list of all events (2C requests and their arrivals) and I is a sub-event list during a single epoch (round). Epsilon and max_iter are the thresholds and the maximum number of iterations for *k_means*, respectively, and T is the time of a single epoch.

## 5. Poisoning Attacks in 2C ML-Based Classification

Most machine learning algorithms are designed to work on a specific issue, known as artificial narrow intelligence (ANI) [41], and little modifications in the operating environment or operational data impact the effectiveness of ML algorithms. Here, we propose two attacks to demonstrate the security issues ML approaches may face. The attacks are channel-independent, and thus the channel characteristics and transmission medium were not considered.

By carefully inserting new examples into the dataset, the attacks aim to alter the behavior of the ML model. The objective of the ML technique is to group data points that are related together or classify them. In a poisoning attack, malicious data points are purposefully added to the dataset in an attempt to affect the results of the ML algorithm. The attacks aim to interrupt the clustering process, the classification process, or both. This can be achieved by fooling the system into forming improper clusters, classifying new examples correctly, or starting new clusters by intentionally grouping poisoned data points.

The proposed attacks are named *attack1* and *attack2*. *Attack1* inserts several poisoning examples that form uncorrelated poisoning data points into the 3D feature plan. The targeted examples were chosen to be in the decision regions between the legitimate clusters. By the decision regions, we mean the regions where the ML algorithms were sensitive to either clustering or classification, such as the regions between two adjacent centroids. To target the examples and the decision regions, we assumed that the attacker had full knowledge of the clustering process. This could be achieved using exploratory attacks. The attack aims to widen the legitimate clusters, and hence the classification will be affected later.

**Figure 3:**
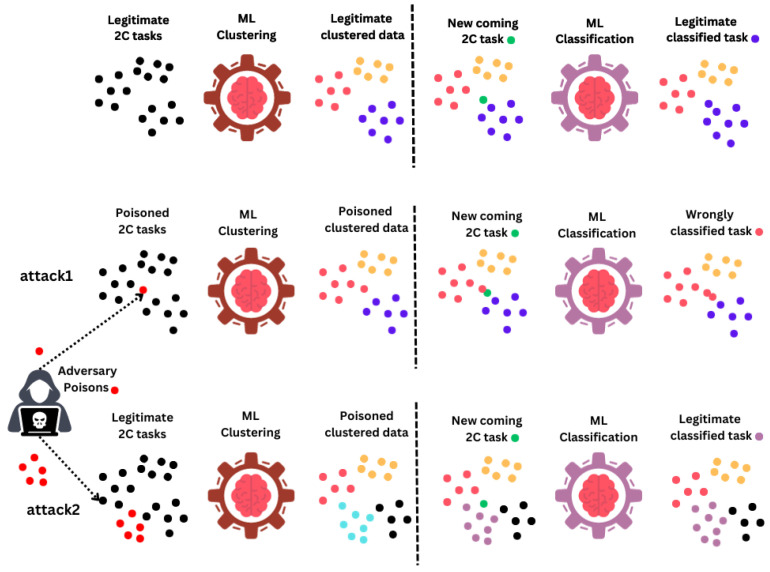
The *attack1* and *attack2* 2D representations.

For *attack2*, the scenario is different. The attacker impersonates a legitimate identity to transmit as a legitimate source. Then, the poisoning examples are grouped (correlated) to form a cluster called a *malicious cluster*. This *malicious cluster* has a mean represented by an example (centroid) located randomly in the 3D features plan and a variance. The purpose of this attack is to fool the whole clustering process. The assigned 2C link may not satisfy the evaluation constraints of the wrongly clustered examples or the next ones for classification. In addition, servers can be overloaded if they process some heavy tasks in terms of computational complexity. Both attacks were simplified in a 2D plane for representation in Figure 3.

**Figure 4:**
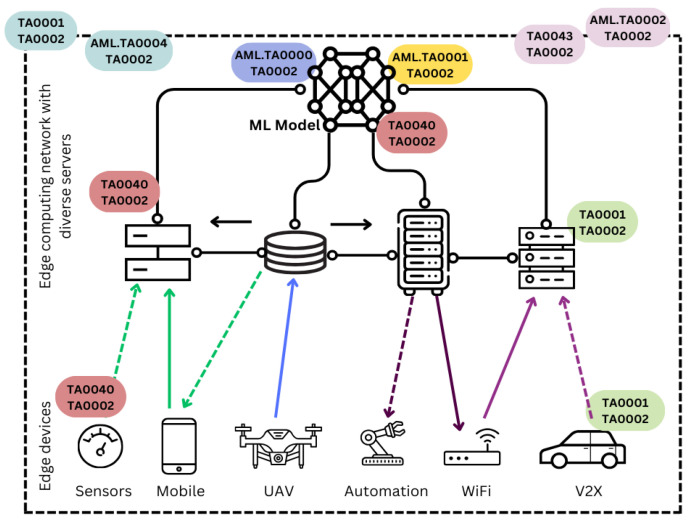
MITRE attacks in the system architecture.

Note that *attack1* targets the features (altering the examples and labels), and thus the attack is said to be feature poisoning. On the other hand, *attack2* targets the clustering itself, and it is said to be cluster poisoning. Both attack examples were launched using *MITRE* [42] tactics and techniques into the system. The launch time instances followed the same distribution (Poisson) as the legitimate sources’ transmission distribution. This was achieved by the combination shown in Figure 4 using cyber security *MITRE matrices* with the tactics listed in Table 3. The execution tactic was performed using the Python technique (T1059.006), where scripts were used to execute all the tactics.

For better understanding, both attacks started by (I) gathering information for future operations using AML.TA0002 on the machine learning model and TA0043 on the network nodes. The next step (II) was to gain access to both the machine learning system and the edge computing model. To achieve this, AML.TA0004 and TA0001 were applied, respectively. After this step, the attacks differentiated. Starting with *attack1*, ( $III_{attack1}$ ) it gained access to the machine learning model using AML.TA0000. After that, ( $IV_{attack1}$ ) the attacker poisoned the dataset using AML.TA0001. The attacker chose the regions between the clusters since full knowledge about the ML model and system was gained. For *attack2*, ( $III_{attack2}$ ) the attacker chose a legitimate source and manipulated its data using TA0040. After that, ( $IV_{attack2}$ ) the source’s manipulated data were injected as poisons using AML.TA0001. All the previous steps for both attacks were launched using Python scripts with the TA0002 tactic. Both attacks’ transmissions followed Poisson distributions with the same parameters as those in the legitimate scenario.

## 6. Poisoning Attack Detection

Anomaly detection (AD) is a data analysis approach that identifies patterns, instances, or observations that differ significantly from normal behavior in a dataset. AD can be accomplished using a variety of statistical, machine learning, or data mining approaches. In our case, because the dataset consisted of several distinct clusters that followed Gaussian distributions, the Gaussian mixture model *(GMM)* [43] was considered to characterize the 2C task features. The GMM is a probabilistic model used for representing complex data distributions as a combination of multiple Gaussian distributions. It assumes that the observed data are generated by a mixture of underlying Gaussian components. Each component in the GMM reflects a Gaussian distribution defined by its mean and covariance. To reflect the overall distribution of the data, the model combines these differentiated Gaussian distributions with mixing coefficients.

The poisoning attack mitigation technique proposed is based on anomaly detection of the poisoning examples. Once they are detected, they are likely to be removed from the dataset. The proposed AD technique is a semi-supervised learning technique used to detect anomalies by modeling the usual behavior of a dataset (reference dataset) and finding data points that vary considerably from this learned normal distribution. As a result, the only possible solution is to have some history of old generated requests by sources representing the 5G and 6G services.

The decision about the anomalous examples is accomplished by using a threshold that represents the maximum 3D position that is far from the cluster’s centroid. Hence, examples that are located far from the clusters’ centroids are said to be anomalous. The other examples with positions near the clusters’ centroids are said to be legitimate. This threshold is determined by the percentile of the GMM’s log-likelihood *score_sample* quantity from the *Scikit-learn* [44] *GMM* class.

For *attack1*, the examples to be monitored are all the dataset examples, while for *attack2*, the monitored examples are the centroids of each cluster, since the aim is to detect the whole malicious cluster. Anomaly detection using the GMM for this attack is named *method1*. For *attack2*, two scenarios might be distinguished: (1) when the malicious cluster is separated well from the legitimate groups and (2) when it intersects with one or more of the legitimate clusters. For this, the *silhouette score* test [45] was employed to first identify the optimum number of clusters the dataset set could be divided into and second, from the system’s history and based on the dataset shape, define the number of legitimate clusters L. Hence, if the *silhouette score* refers to a greater number of clusters than L, then anomaly detection is run to detect the malicious centroid. If the detected malicious cluster is found to be intersecting with legitimate clusters, then *quantile-quantile* (Q-Q) plots [46] are used to separate it. Anomaly detection using the GMM with the *silhouette score* and Q-Q plots is named *method2*.

To summarize, the following apply to (A) application of the detection for *attack1*:

1. Obtain the optimum number of clusters k in the data using the silhouette score test.
2. Train the GMM model offline with all the examples used as history.
3. Check if the model has intersecting clusters using Q-Q plots for each group. If the line of the plot is not straight compared with the Gaussian distribution reference, then the group is detected as not being Gaussian distributed. Hence, a possible intersection is present, and k is incremented by the amount of intersections detected.
4. Fit the GMM on the model.
5. For each example in the dataset, its 3D position log-likelihood is compared to the threshold. If it is greater than the threshold, then it is anomalous (i.e., the example located far from the centroid of the associated cluster). If its 3D position log-likelihood is lower than the threshold, then the example is said to be legitimate (i.e., it is near the cluster centroid).

The following apply to (B) application of the detection for *attack2*:

1. Obtain the optimum number of clusters k in the data using the silhouette score test.
2. Train the GMM model offline with the clusters’ centroids only used as history.
3. Check if the model has intersecting clusters using Q-Q plots for each group. If the line of the plot is not straight compared with the Gaussian distribution reference, then the group is detected as not being Gaussian distributed. Hence, a possible intersection is present, and k is incremented by the amount of intersections detected.
4. Fit the GMM on the model.
5. For each centroid in the dataset, its 3D position log-likelihood is compared to the threshold. If it is greater than the threshold, then it is anomalous (i.e., the centroid is located at an anomalous position based on the history). If its 3D position log-likelihood is lower than the threshold, then the centroid is said to be legitimate (i.e., it is located in a normal position based on the history).

## 7. Simulation Results

### 7.1. Constraints

The simulation tools were *Jupyter Notebooks* with *Python3* scripts. We avoided using some real traffic generators, real environment simulators, and modeling the 5G infrastructure because of the distortions modeled under these scenarios. This study aimed to investigate the effects of poisoning attacks on the classification of 2C requests. With real wireless and wired channel distortions, the poisoning effects may not have been analyzed. As a result, environment modeling was not considered.

### 7.2. Evaluation Tools

The classification algorithm performance was evaluated using three metrics: the amount of processing time, the number of unaccepted requests, and the number of used servers. The same metrics were used to evaluate the ML classification under attack and after applying the mitigation techniques. The processing time is the time a link server takes to process the task, and it is the time spent by both the CPU and GPU processing units. This metric was used to evaluate the algorithm for the computing part only. The number of unaccepted requests represents the number of requests assigned to a link server with either higher latency, lower reliability, or lower computational complexity. This metric was used to evaluate both the communication and computing parts of the algorithm. The number of used servers represents the number of link servers that were not idle during the whole process. If a link server is assigned to one single request, then it is said to be used.

### 7.3. Results

In this section, we provide the results of the simulated 2C task classification, the impact of *attack 1* and *attack 2* on this classification, and the results of the application of *method 1* and *method 2* for attack detection (and mitigation).

For reference and comparison with our methods, the 2C requests assigned to the servers (the serving 2C links) in a round robin fashion [47] were considered. Since the ML technique is a real-time classifier and has to be data-empty, there was no way to cluster or classify using the ML algorithm in the first epoch or round. For that, 1000 extra-classified requests were used as an offline initial state to the ML data. These examples were not considered in the results. The dataset was fed to the ML classification system. The dataset (The 1000 examples without consideration of the extra ones was preprocessed using the *PowerTransformer* scaler with the *Box-Cox* method followed by *MinMaxTransformer* (with min = 0, max = 100) was from the *Scikit-learn* library [44]. The *PowerTransformer* transforms the data in each axis to be Gaussian-like distributed. In addition, the *MinMaxTransformer* gives a range of data between 0 and 100, and this made the *k-means* and *k′-NN* metrics (3D Euclidean distance) fair between all the dataset samples. This choice also came from the intention to set the third axis (third dimension) of the Gaussian-like distributed dataset. Keep in mind that this axis was simulated using a uniform distribution. This ensured that all the 3D dimensions of the dataset followed the same distribution type and range (i.e., Gaussian shape with a range [0, 100]).

**Figure 5:**
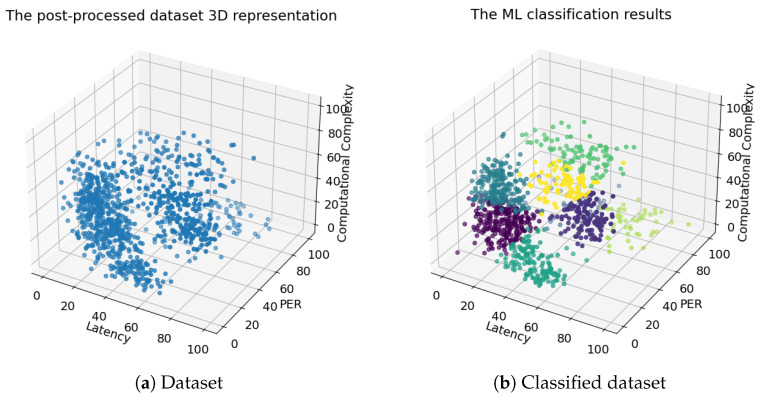
Attack 3D representations.

After serving all the 2C requests, the ML classification converged to eight distinct clusters, as shown in Figure 5. Since the usage pattern of 5G services is stochastic, the number of needed servers to serve the 2C tasks could not be defined. As a result, the silhouette score test was used to determine the optimum number of clusters. This was accomplished by choosing the number of servers (clusters) that had the highest silhouette score among all the possible combinations. We limited our consideration to only eight servers, while for future work, more servers will be considered.

**Figure 6:**
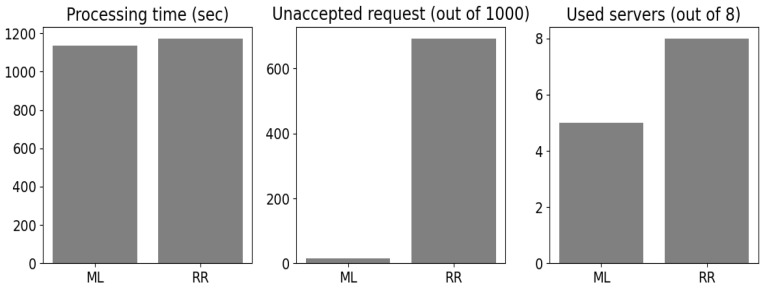
Machine learning (ML) vs. round robin (RR) results.

The 2C link servers were chosen as shown in Table 2. We considered the CPU and GPU computing for the servers (computing part) and diverse radio and wired channels with different propagation conditions that defined the PER and latency introduced by each link (communication part). The servers were assumed to be buffer unlimited (i.e., no task was lost). The benefits of the ML classifiers compared with the simple round robin technique are shown in Figure 6.

The processing time represents the time all servers took to process the 2C tasks. The server CPUs were assumed to behave as round robin schedulers with parallel GPU processing. The 2C request was said to not be accepted if it was allocated to a link server that did not fulfill the minimum evaluation constraints of this task’s service, while the quantity of the used server was the total number of servers used by the allocation technique. The hybrid *k-means/k′-NN* method showed rather high gains against the round robin method. Even though only five servers were enabled to serve the eight clusters, around 40 seconds (40 s) less time was observed compared with the round robin method with eight servers. In addition, only 14 tasks were not accepted in terms of the evaluation constraints guaranteed by the assigned link server compared with 693 for the round robin method. Because the main focus of this paper is the security aspects of ML, only the round robin method was compared to the proposed algorithm to show that AI is optimal for assisting edge intelligence.

**Figure 7:**
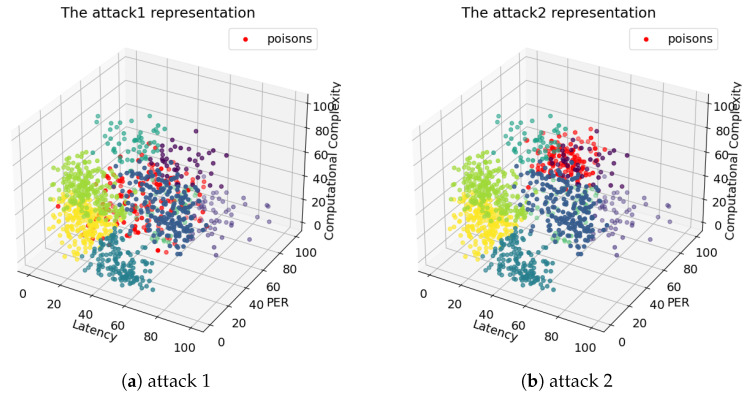
Attack 3D representations.

Based on the MITRE techniques and tactics discussed earlier, we launched the attacks on virtual machines that ran the sources and control system infrastructure. This section discusses the numerical results obtained after the attacks were successfully launched. We set the rate of success of exploratory attacks to 0.7. This means that in 70% of the attacker’s attempts, the poisoning examples were located in the decision regions, while the rest (30%) were randomly located in the 3D plane. Visualizations of both types of attacks are shown in Figure 7.

**Figure 8:**
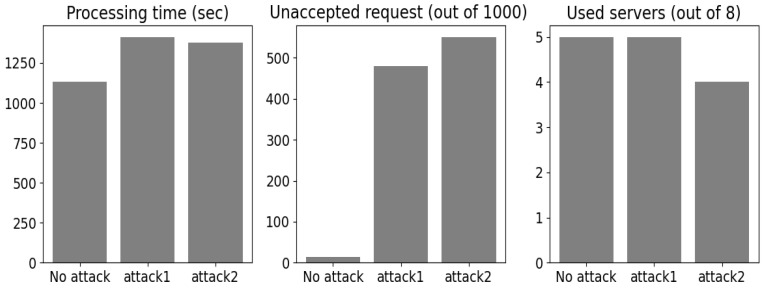
Attacks’ effects.

The number of injected poisons was equal to the average population between all the clusters. Both attacks showed dramatic degradation in ML performance. The serving time increased by 278 and 241 seconds after introducing *attack1* and *attack2*, respectively (Figure 8). In addition, for attack2, only four servers were active, which means the other servers were being overloaded, and this demonstrated the increase in processing time with the grouped poisoning examples. Furthermore, for both attacks, we observed a higher number of tasks that were not accepted in terms of evaluation constraints. The results refer to 479 and 551 tasks not accepted for *attack1* and *attack2*, respectively.

**Figure 9:**
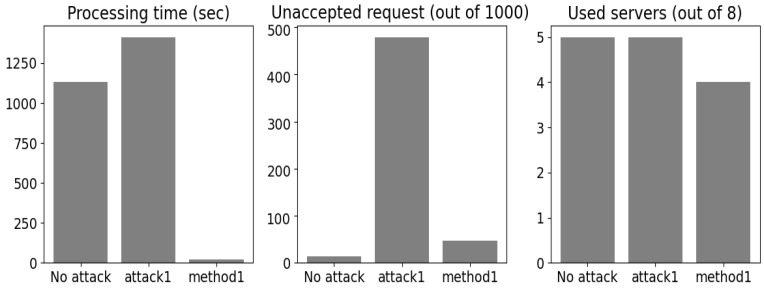
*Method 1* results.

To launch anomaly detection as a mitigation technique, a history was created as a training (reference) dataset, since the method involves semi-supervised learning. The history consisted of all the possible 5G services defined by ETSI in “(2022-05)1683GPP TS 23.501 version 17.4.0” release 17. All the services were created and modeled in the same way as the chosen ones in the previous step. Once the history was created, *method1* was launched on *attack1*. The obtained results are shown in Figure 9. The decision about anomalies was made based on the percentile of the log-likelihood score of the *GMM*. The score that maximized the precision (true positives over true positives and false positives combined) was −14.52 with a percentile of 9. The choice of precision was justified as follows. Since the main objective was to detect anomalies without losing legitimate requests, the minimization of false positives (FP), also called false alarms, was critical, and hence our maximizing the precision.

Anomaly detection using *GMM* brought about a quite powerful mitigation solution. The unaccepted tasks based on the evaluation constraints dropped to near the optimal value (no attack scenario) compared with the *attack1* scenario. However, the drawback is that the solution forced the ML to enable four servers with higher computational capabilities. This means more power was consumed, and the processing time was rather low compared with the optimal solution. In addition, only 31 requests were detected as false positives (FP), also called false alarms, and as a result these requests were lost.

**Figure 10:**
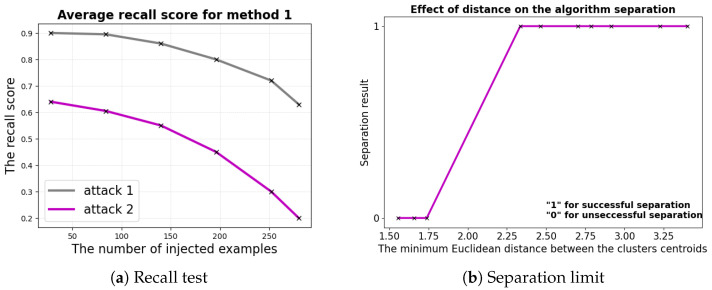
*Method 2* limitations.

This method was devoted only to *attack1*, since the *attack2* examples appeared to be legitimate under the method’s constraints and could not be detected. The recall score (the ratio of true positives to true positives and false negatives combined) dropped when using *method1* as a mitigation solution for *attack2*. The choice this time was the recall because the main goal was to not miss the malicious cluster examples (false negatives (FNs)) when the cluster was distinguished well from the others. To gain insight into this, multiple simulations were run for different numbers of injected poisons, and the recall test results were obtained (see Figure 10a).

Since the anomaly detection had a history of all the 5G services, the attacker may have matched a centroid where a 5G service was originally located nearby, and as a result, the technique mistakenly detected these examples. For this, the *silhouette score* test was introduced to omit one of the clusters obtained by the *GMM*. But the history now is different. Since the aim was to detect and omit the whole malicious cluster, only the centroids of the groups were present in the history.

To complete this, as mentioned before, Q-Q plots were used in case the clusters intersected. The Q-Q plots were obtained for each obtained group with the *GMM*. If the line was not straight about the normal (Gaussian) theoretical quantiles, this means that the group was not Gaussian-like. And since all the services were modeled as being 3D Gaussian, the cluster consisted of intersecting clusters. At some point, where the intersected clusters’ centroids were quite near to each other, the separation process failed. The results of this process are presented in Figure 10b.

The minimum Euclidean distance was unit-free since it was a position in the 3D plane. Note that even if separation was performed, false positives and false negatives might have been present. The results presented in the figure illustrate that the recall test was enhanced but was still worse than the *attack1* mitigation.

**Figure 11:**
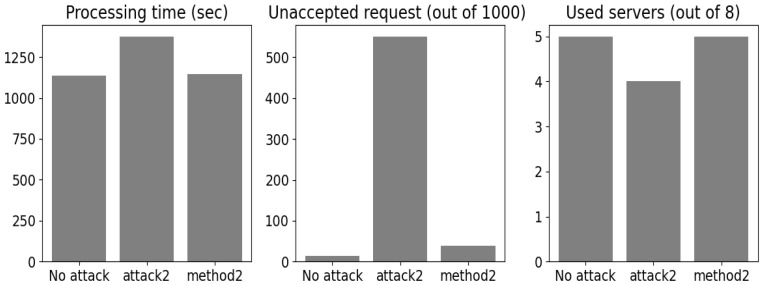
*Method 2* results.

As a result, launching *method2* on *attack2* yielded the results shown in Figure 11. The method recovered on average the same clustering scheme with 78 false negatives (FNs, or missed detections) and 33 FPs (false alarms). These missed detection examples and the lost ones introduced 10 more seconds of processing time compared with the optimal scenario. In addition, 24 more 2C requests were unacceptable regarding the evaluation constraints ensured by the assigned link servers.

The simulation parameters used are summarized in Table A2. In addition, the servers activated by each step are summarized in Table A1.

### 7.4. Detection Complexity

Since anomaly detection is a semi-supervised learning technique, it can be trained offline using appropriate tools such as supercomputers. The detection of each example or centroid required only one real comparison. As a result, the algorithm was of a linear complexity. The number of comparisons needed equaled the number of examples desired to be checked.

## 8. Conclusions

Throughout this article, a hybrid *k-means/k′-NN* technique was presented to assist with near-optimal 2C task classification in edge computing for 5G and beyond communication systems. The technique outperformed the classical round robin scheduling technique in terms of the processing time of the 2C tasks, the fulfillment of constraints for the assigned link server to each task, and the number of used servers. After that, two attack types, named *attack1* and *attack2*, were proposed to weaken the performance of the ML method. Both methods showed down-degradation in the ML technique. Higher unaccepted 2C tasks regarding the evaluation constraints were observed, and longer processing times were observed under the presence of these attacks. The later attack successfully launched cluster poisoning, where the ML classification scheme changed completely and the servers became overloaded by the examples assigned to other omitted clusters. Finally, to mitigate these attacks, two anomaly detection methods were proposed. Each one was devoted to a single attack type due to the limitations they faced. To mitigate *attack1*, more power was needed where the end server had higher computational capability, but this ensured the minimum QoS needed to serve the tasks under this attack. For *attack2*, extra statistical tools were used. Both mitigation techniques showed good detection and recovery of the classification system, where the processing time recovered to near the optimal scenario. In addition, lower unaccepted 2C requests in terms of evaluation constraints were observed when applying these solutions.

For future considerations, a complete system will be studied, and link servers with higher diversity will be addressed. In addition, the propagation conditions in both the radio and wired media will be studied. A full real environment with real network characteristics will be modeled. Hence, the impact of poisoning attacks in real-world scenarios will be covered. Moreover, how the channel can affect the poisons and how the attacker has to be aware of the channel conditions will be analyzed. Furthermore, the ML technique has to be more optimized and compared to the state-of-the-art techniques. The exploration phase of attacks will be analyzed as well.

## Figures and Tables

**Table 1:** Attack classification in AML with 2C task classification relevance.

Attack	Description	Example for 2C Task Classification
Exploratory (Inference) Attacks	Seek to discover the model mechanism using the training data and then imitate the model by building a surrogate model. This is realized by probing the input and the outputs.	Build a surrogate model and use it to find its decision boundaries. This can be used later to launch attacks near these decision boundaries.
Membership Inference Attacks MIA	The adversary aims to determine if a given data sample is a member of the training data, and then the design of the attack can be more successful.	For example, the attacker aims to determine whether a service is used for clustering. This allows the attacker to launch a more sophisticated attack.
Evasion (Adversarial) Attacks	The aim is to manipulate the input test data to fool the model into making the wrong decision. An evasion attack determines the samples that the target classifier is likely to misclassify.	Inject adversarial examples to the 2C task allocation ML model to fool the classifier.
Spoofing Attacks	The adversary generates synthetic data samples from scratch rather than adding perturbations to the real ones.	Spoof the ML model and use it to classify the attacker data as high QoS tasks, hence overloading the dedicated VMs for high QoS tasks.
Causative (Poisoning) Attacks	The aim is to manipulate the training process of models by injecting weaknesses, such as altering the training data of the model. Provides erroneous training data samples to reduce the reliability of the classifier.	Target cluster poisoning by injecting a new cluster, which will fool the classifier and reduce the allocation system performance.
Trojan (Backdoor) Attacks	Combination of evasion and causative attacks, where the adversary injects triggers (backdoors) into training data and then activates them for some input samples in test time.	This can be used, for example, to target specific types of data such as mission-critical data, and these data will be wrongly classified as VMs of 2C link with lower QoS.

**Table 2:** The 2C links, attributes, and representation.

i	End-to-End Latency	Reliability	Computational Capability	Representation
0	Low	Low	Low	000
1	Low	Low	High	001
2	Low	High	Low	010
3	Low	High	High	011
4	High	Low	Low	100
5	High	Low	High	101
6	High	High	Low	110
7	High	High	High	111

**Table 3:** MITRE tactics used for attacking the ML system.

ID	Name	Description	Type
AML.TA0002	Reconnaissance	Gather information for future operations	ATLAS
TA0043	Reconnaissance	Gather information for future operations	ATT&CK
AML.TA0004	Initial access	Enter into the ML system	ATLAS
TA0001	Initial access	Enter into the edge-computing network	ATT&CK
AML.TA0000	ML model access	Enter into the ML model	ATLAS
TA0002	Execution	Run malicious code	ATT&CK
AML.TA0001	ML attack staging	Poison the data	ATLAS
TA0040	Impact	Manipulate the data	ATT&CK

## Data Availability

Data is unavailable due to privacy.

## Appendix A

**Table A1:** The servers activated by each step.

Case	Server Number	Binary Representation	Computational Capability (GFLOPs)
Round robin	8	1.’000’, 2.’001’, 3.’010’, 4.’011’, 5.’100’, 6.’101’, 7.’110’, 8.’111’	1.1450, 2.4350, 3.1450, 4.4350, 5.1450, 6.4350, 7.1450, 8.4350
Optimal ML	5	1.’101’, 2.’110’, 3.’000’, 4.’001’, 5.’010’	1.4350, 2.1450, 3.1450, 4.4350, 5.1450
ML attack1	5	1.’001’, 2.’010’, 3.’000’, 4.’101’, 5.’110’	1.4350, 2.1450, 3.1450, 4.4350, 5.1450
ML attack2	4	1.’101’, 2.’000’, 3.’110’, 4.’001’	1.4350, 2.4350, 3.1450, 4.1450, 5.1450
ML attack1, method1	4	1.’001’, 2.’111’, 3.’011’, 4.’101’	1.4350, 2.4350, 3.4350, 4.4350
ML attack2, method2	5	1.’101’, 2.’110’, 3.’000’, 4.’001’, 5.’010’	1.4350, 2.1450, 3.1450, 4.4350, 5.1450

**Table A2:** Simulation parameters.

Parameter Symbol	Quantity
k′	50
C	random
max_iter	10,000
epsilon	1 × 10^−10^
T	10
